# Bidirectional variation in glutamate efflux in the medial prefrontal cortex induced by selective positive and negative allosteric mGluR5 modulators

**DOI:** 10.1111/jnc.14290

**Published:** 2018-02-12

**Authors:** Sarah N. Isherwood, Trevor W. Robbins, Jeffrey W. Dalley, Anton Pekcec

**Affiliations:** ^1^ Boehringer Ingelheim Pharma GmbH & Co. KG, Div. Research Germany Biberach an der Riss Germany; ^2^ Behavioural and Clinical Neuroscience Institute University of Cambridge Cambridge UK; ^3^ Department of Psychology University of Cambridge Cambridge UK; ^4^ Department of Psychiatry University of Cambridge Cambridge UK

**Keywords:** glutamate, impulsivity, mGluR5, NMDA receptors, prefrontal cortex

## Abstract

Dysregulation of prefrontal cortical glutamatergic signalling *via* NMDA receptor hypofunction has been implicated in cognitive dysfunction and impaired inhibitory control in such neuropsychiatric disorders as schizophrenia, attention‐deficit hyperactivity disorder and drug addiction. Although NMDA receptors functionally interact with metabotropic glutamate receptor 5 (mGluR5), the consequence of this interaction for glutamate release in the prefrontal cortex (PFC) remains unknown. We therefore investigated the effects of positive and negative allosteric mGluR5 modulation on changes in extracellular glutamate efflux in the medial PFC (mPFC) induced by systemic administration of the non‐competitive NMDA receptor antagonist dizocilpine (or MK801) in rats. Extracellular glutamate efflux was measured following systemic administration of the positive allosteric mGluR5 modulator [S‐(4‐Fluoro‐phenyl)‐{3‐[3‐(4‐fluoro‐phenyl)‐[1,2,4]‐oxadiazol‐5‐yl]‐piperidin‐1‐yl}‐methanone] (ADX47273; 100 mg/kg, p.o.) and negative allosteric mGluR5 modulator [2‐chloro‐4‐{[1‐(4‐fluorophenyl)‐2,5‐dimethyl‐1H‐imidazol‐4‐yl]ethynyl}pyridine] (RO4917523; 0.3 mg/kg, p.o.), using a wireless glutamate biosensor in awake, freely moving rats. The effect of MK801 (0.03–0.06 mg/kg, s.c.) on mPFC glutamate efflux was also investigated in addition to the effects of MK801 (0.03 mg/kg, s.c.) following ADX47273 (100 mg/kg, p.o.) pre‐treatment. ADX47273 produced a sustained increase in glutamate efflux and increased the effect of NMDA receptor antagonism on glutamate efflux in the mPFC. In contrast, negative allosteric mGluR5 modulation with RO4917523 decreased glutamate efflux in the mPFC. These findings indicate that positive and negative allosteric mGluR5 modulators produce long lasting and opposing actions on extracellular glutamate efflux in the mPFC. Positive and negative allosteric modulators of mGluR5 may therefore be viable therapeutic agents to correct abnormalities in glutamatergic signalling present in a range of neuropsychiatric disorders.

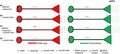

**Abbreviations used**
5‐CSRTT: 5‐choice serial reaction time task
ADHD: attention‐deficit hyperactivity disorder
ADX47273: S‐(4‐Fluoro‐phenyl)‐{3‐[3‐(4‐fluoro‐phenyl)‐[1,2,4]‐oxadiazol‐5‐yl]‐piperidin‐1‐yl}‐methanone
GABA: Gamma‐aminobutyric acid
i.v.: intravenous
mGluR2/3: metabotropic glutamate receptor 2/3
mGluR5: metabotropic glutamate receptor 5
mPFC: medial prefrontal cortex
NMDA: N‐methyl‐D‐aspartate
p.o.: perioral
PFA: paraformaldehyde
PFC: prefrontal cortex
RO4917523: 2‐chloro‐4‐{[1‐(4‐fluorophenyl)‐2,5‐dimethyl‐1H‐imidazol‐4‐yl]ethynyl}pyridine
s.c.: subcutaneous

Dysregulation of prefrontal glutamatergic signalling is hypothesized to underlie impaired cognitive functioning in a number of neuropsychiatric disorders, including schizophrenia, attention‐deficit hyperactivity disorder and drug addiction (Deakin *et al*. 1989; Goff and Coyle 2001; Konradi and Heckers 2003; Lindsley *et al*. 2006; Kalivas and Volkow 2011; Archer and Garcia 2016). In rodents and non‐human primates, as well as in humans, N‐methyl‐D‐aspartate (NMDA) receptors contribute to cognitive functions dependent on the prefrontal cortex (PFC), including working memory (Aultman and Moghaddam 2001; Karlsgodt *et al*. 2011; Driesen *et al*. 2013; Barker and Warburton 2015; Wang and Arnsten 2015) and executive attention (Malhotra *et al*. 1996; Castner and Williams 2007; Graybeal *et al*. 2012).

We recently reported that positive allosteric mGluR5 modulation decreases impulsive responding in the 5‐choice serial reaction time task (5‐CSRTT) and attenuates the disruptive effects of NMDA receptor antagonism on this task (Isherwood *et al*. 2015). Although the neural mechanisms underlying these effects remain unknown, optimal performance on this task depends on the PFC and glutamatergic signalling from this region to a variety of cortical and subcortical structures (Robbins 2002). Supporting a putative locus within the PFC for these effects, NMDA receptor antagonists increase the activity of pyramidal neurons in this region (Suzuki *et al*. 2002; Jackson *et al*. 2004; Lecourtier *et al*. 2007) and locally increase glutamate release (Moghaddam *et al*. 1997; Adams and Moghaddam 1998; Moghaddam and Adams 1998; Abekawa *et al*. 2003; Lorrain *et al*. 2003; Ceglia *et al*. 2004). NMDA receptor antagonists also increase impulsivity in the 5‐CSRTT (Amitai *et al*. 2007; Fletcher *et al*. 2011; Higgins *et al*. 2003b; Isherwood *et al*. 2015; Oliver *et al*. 2009; Paine *et al*. 2007; see Amitai and Markou 2010 for review), which may result from increased synaptic availability of glutamate at non‐NMDA receptors (Moghaddam *et al*. 1997; Moghaddam and Adams 1998). Furthermore, suppressing glutamate release or pharmacologically blocking non‐NMDA receptors reduces impulsivity and restores the cognitive impairment associated with NMDA receptor antagonism (Moghaddam *et al*. 1997; Moghaddam and Adams 1998; Pozzi *et al*. 2011).

Previous studies have shown that activation of mGluR5 enhances NMDA receptor function (Campbell *et al*. 2004; Homayoun *et al*. 2004; Liu *et al*. 2008; Rosenbrock *et al*. 2010; Stefani and Moghaddam 2010). Moreover positive allosteric modulation of mGluR5 reverses MK801‐induced excessive activity and burst firing of neurons in the PFC of freely moving rats (Lecourtier *et al*. 2007). However, it remains unknown whether NMDA receptors interact with mGluR5 to regulate mechanisms affecting glutamate release in the PFC. Thus, although orthosteric mGluR5 agonists increase aspartate and glutamate release in the central nervous system (Thomas *et al*. 2000; Park *et al*. 2004; Musante *et al*. 2008), no *in vivo* study to date has investigated whether mGluR5 PAMs affect the elevation in PFC glutamate release caused by NMDA receptor antagonists. Since positive allosteric mGluR5 modulation putatively activates GABA‐ergic interneurons in the PFC (Chu and Hablitz 1998; Lecourtier *et al*. 2007) which in turn act to inhibit glutamatergic pyramidal neurons in this region (Krystal *et al*. 2003), we hypothesized that mGluR5 PAMs may reverse the cognitive impairing effects of NMDA receptor antagonists by attenuating glutamate release in the PFC.

In this study we investigated this hypothesis using wireless glutamate biosensor technology in awake, freely moving rats to assess the effects of a selective mGluR5 PAM and a selective mGluR5 NAM on the extracellular concentration of glutamate in the mPFC. Changes in extracellular glutamate concentration were measured following the systemic administration of the NMDA receptor antagonist MK801, the positive allosteric mGluR5 modulator [S‐(4‐Fluoro‐phenyl)‐{3‐[3‐(4‐fluoro‐phenyl)‐[1,2,4]‐oxadiazol‐5‐yl]‐piperidin‐1‐yl}‐methanone] (ADX47273; Isherwood *et al*. 2015; Liu *et al*. 2008) and the negative allosteric mGluR5 modulator [2‐chloro‐4‐{[1‐(4‐fluorophenyl)‐2,5‐dimethyl‐1H‐imidazol‐4‐yl]ethynyl}pyridine] (RO4917523; Isherwood *et al*. 2015; Lindemann *et al*. 2015). Subsequently, we investigated whether ADX47273 modulates glutamate efflux induced by systemic MK801 administration.

## Materials and methods

### Subjects

Male Wistar Han rats (RRID:RGD_2308816), weighing 250–280 g on arrival (Charles River, Germany), were initially housed in groups of four under a 12 h light/dark cycle, at ca. 22°C, with food and water available *ad libitum* and environmental enrichment consistent of a plexiglas tube and gnawing sticks. At least 1 week's acclimatization was given before surgery, during which time all rats were habituated with daily handling. Following surgery to implant the glutamate biosensor, all rats were individually housed for the remainder of the study. The experiments were conducted between the hours of 07 : 00 and 15 : 00 with at least a week washout period between each experiment. All experimental procedures were authorized by the Local Animal Care and Use Committee (VVH 12‐029 & 15‐002) in accordance with local animal care guidelines, AAALAC regulations and the USDA Animal Welfare Act. The study was not preregistered.

### Drugs and drug bioavailability

The tool compounds, [S‐(4‐Fluoro‐phenyl)‐{3‐[3‐(4‐fluoro‐phenyl)‐[1,2,4]‐oxadiazol‐5‐yl]‐piperidin‐1‐yl}‐methanone] (ADX47273) and [2‐chloro‐4‐{[1‐(4‐fluorophenyl)‐2,5‐dimethyl‐1H‐imidazol‐4‐yl]ethynyl}pyridine] (RO4917523), were synthesized and supplied by Boehringer Ingelheim Pharma GmbH & Co. KG (Germany). Both compounds were dissolved in 10% Tween 80 (0.1% v/v) and 90% natrosol (0.5% v/v) and administered orally (p.o.) at 2 ml/kg. (+)‐MK801 hydrogen maleate was purchased from Sigma‐Aldrich (Taufkirchen, Germany), dissolved in saline (0.9%) and administered subcutaneously (s.c.) at 1 mL/kg after adjustment to pH 7.4. The doses of ADX47273, RO4917523 and MK801 were selected based on previously published behavioural data (Isherwood *et al*. 2015). Plasma levels of MK801, ADX47273 and RO4917523 were measured to determine drug bioavailability and to ensure suitable drug exposures were attained in Wistar Han rats (compared with Lister‐Hooded rats used previously, Isherwood *et al*. 2015). Blood samples were taken 15 min after MK801 (0.03 mg/kg, s.c.), 1 h 30 min after ADX47273 (100 mg/kg, p.o.) and 2 h after RO4917523 (0.1 mg/kg, p.o.) administration *via* the sublingual vein. Rats were lightly anaesthetized under 5% isoflurane (with oxygen) until all reflex responses were absent. The tongue was gently extended and, using a sharp needle, the sublingual vein was punctured. Whole blood was collected (enough to provide 100 μL plasma) in potassium EDTA pre‐conditioned vials (Kabe Labortechnik GmbH, Germany), to avoid blood coagulation, and stored on ice until preparation of the plasma by centrifugation. The blood samples were centrifuged no more than 30 min after blood sampling at ca 2000 g for 10 min at 4°C. The plasma supernatant was subsequently collected and frozen at −20°C until analysis. Drug levels were measured using mass spectroscopy.

### Surgery

For glutamate sensor guide cannulae implantation, rats were anaesthetized with ‘medetomidine/ midazolam/ fentanyl (MMF)’ (1 mL/kg, i.m; medetomidine hydrochoride (150 μg/kg; Komtur Apotheke, Germany), midazolam (2 mg/kg; HEXAL AG, Germany) and fentanyl (5 μg/kg; Janssen, Germany), dissolved in saline (0.9%), with co‐administration of the analgesic meloxicam (0.5 mg/kg, 0.1 mL s.c., Boehringer Ingelheim, Germany). MMF was chosen for sensor guide cannulae implantation as (i) anaesthesia is fully reversible, (ii) well tolerated by rats with low incidence of adverse events or surgery‐related complication, (iii) the oro‐nasal inhalation mask required for isoflurane anaesthesia interferes sterically with the head caps required to protect the guide cannulae and sensor system. Rats were secured into a stereotaxic frame (David Kopf Instruments, USA) fitted with atraumatic ear bars. The incisor bar was adjusted to ensure a flat skull position. Local anaesthetic (bupivacain, 0.5%; JenaPharm^®^, Germany) was applied to the surgical wound for 2–3 min before exposing the skull. Using standard stereotaxic techniques, glutamate sensor guide cannulae (BAS Rat Cannula, Pinnacle Technology Inc., Lawrence, KS, USA) were implanted unilaterally directly above the left mPFC and secured to the skull with bone screws (Pinnacle Technology Inc.) and dental cement (PermaCem Automix; DMG, Germany). The following coordinates were used: AP +2.7 or 3.2 mm; ML −0.5 mm; DV −1.2 mm, relative to dura and bregma (Paxinos, George; Watson, Charles. (1997). The Rat Brain in Stereotaxic Coordinates. London: Academic Press). Upon completion of surgery, MMF anaesthesia was antagonized with 3 mL/kg, s.c, 750 μg/kg atipamezole hydrochloride (Zoetis, Germany) and 0.2 mg/kg flumazenil (Hameln, Germany), dissolved in 0.9% saline. Rats also received 0.1 mL meloxicam analgesic for 2 days post‐surgery and a prophylactic antibiotic (marbofolxacin, 1%, Germany) administered 0.2 mL/kg, s.c. for 1 week. Rats recovered for at least 1 week before the experiments commenced.

### Glutamate biosensor

Extracellular glutamate efflux in the mPFC was measured using a wireless glutamate biosensor system (Pinnacle Technology Inc.). Each sensor was constructed with a platinum‐iridium electrode coated with glutamate oxidase, which catalyses the breakdown of glutamate to α‐ketoglutarate and hydrogen‐peroxide (H_2_O_2_). The sensor was additionally coated with ascorbate oxidase to catalyse the breakdown of ascorbic acid, thereby reducing interference. The oxidation current (nA) of H_2_O_2_ provided an index of the extracellular concentration of glutamate and was signalled wirelessly *via* Bluetooth^®^ to a control computer.

### Glutamate sensor calibration and implantation

Following recovery from surgery, and approximately 24 h before the start of the experiment, each sensor was calibrated *in vitro* to confirm the sensitivity of the recording system to glutamate. Once calibrated, the sensors were implanted. Experimentation and glutamate recordings started approximately 12 h later. The procedure of calibration involved immersing the sensor in a 20 mL solution of a magnesium‐ and calcium‐free phosphate‐buffered saline (PBS; Gibco^®^ by life technologies, Germany), diluted 1 : 10 and adjusted on a magnetic stirrer to pH 7.4 and 37°C. The sensor was equilibrated for 1 h until the rate of decline in the oxidation current was < 0.2 nA/5 min. At this point, the current was recorded for an additional 10 min as a baseline period. A fresh calibration stock solution of 5 mM glutamate was made prior to calibration (L‐glutamic acid, Roth, Germany). Forty microlitre aliquots of the glutamate stock solution were added three times, every 5 mins, into the PBS solution. In addition, 50 μL of L‐(+)‐ascorbic acid (100 mM) was added to the PBS solution. The final concentration of ascorbic acid of 250 μM was in excess of the estimated concentration within the brain (40‐60 μM; (Tsai *et al*. 1996). Sensors which responded to ascorbic acid (> 1 nA increase in current) were discarded.

Following calibration, rats were lightly anaesthetized with isoflurane (5% in oxygen). The dummy cannulae were removed and the sensors were implanted and attached to a potentiostat powered by a 3V lithium battery (Energizer^®^, Germany). Once awake, rats were returned to the animal holding room until the following day when the experiment began.

On the day of the experiment, all rats were habituated to the testing room for at least 1 h with the recording experiment taking place in the rats’ home cage. Recordings commenced 10 min before drug administration (baseline) and continued for 2–3 h after drug administration. A largely within‐subjects design was used since preliminary experiments indicated a high individual variability in glutamate efflux evoked by NMDA receptor antagonism. Drug doses and pre‐treatment times were chosen on the basis of previously published behavioural and pharmacokinetic data (Isherwood *et al*. 2015). To ensure that the bioavailability of compounds did not differ between rat strains (i.e. between Lister‐hooded rats used in previous behavioural studies (Isherwood *et al*. 2015) and Wistar Han rats used in the present studies), plasma levels of MK801, ADX47273 and RO4917523 were measured in a separate cohort of Wistar Han rats (Table 1).

**Table 1:** Mean plasma levels following the administration of 0.03 mg/kg MK801 (15‐min post‐administration), 100 mg/kg ADX472723 (1‐h and 30‐min post‐administration) and 0.1 mg/kg RO4917523 (2‐h post‐administration) in Wistar Han rats

	Mean plasma concentration (nM); Wistar Han	Mean plasma concentration (nM); Lister‐hooded
MK801 (0.03 mg/kg; s.c.)	7.64 ± 0.1	6.74 ± 0.1
ADX47273 (100 mg/kg; p.o.)	1740.0 ± 176.2	2550 ± 210.0 (Isherwood et., 2015)
RO4917523 (0.1 mg/kg; p.o.)	72.4 ± 4.77	25 ± 0^a^ (Isherwood *et al*. 2015)

Values represent mean ± SEM (*n *=* *3 per group).

a *n *=* *1 (two samples below detectable range).

For comparison, mean plasma levels of 0.03 mg/kg MK801 (10‐min post‐administration) in Lister‐hooded rats are also shown.

### Experimental outline

**Figure 1:**
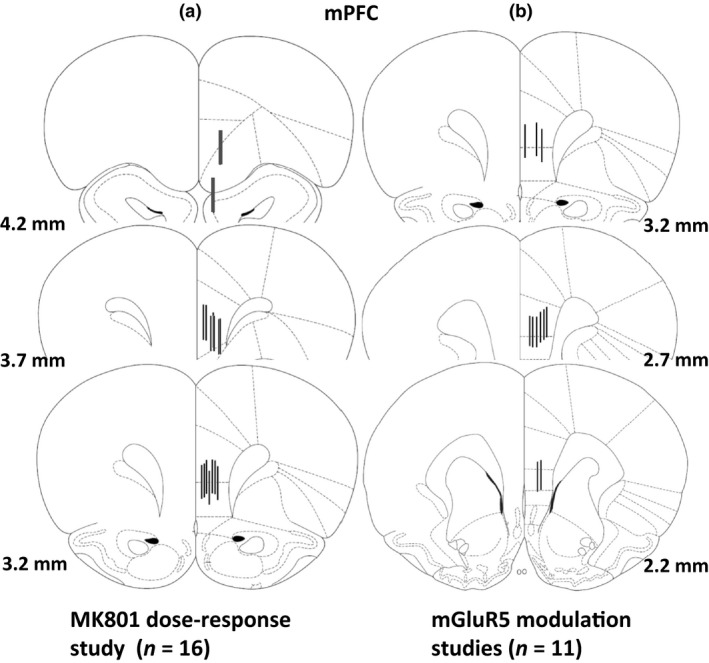
Schematic diagrams of coronal rat brain sections depicting reconstructed biosensor placements within the mPFC. Black lines represent the glutamate‐sensitive region of each sensor. Coordinates are mm forward of bregma. (a) Sensor placements for MK801 dose‐response experiment (*n* = 14 rats; two rats were excluded because of probe placements outside of the mPFC (shown in red). (b) Sensor placements for mGluR5 modulation experiments (*n *=* *11 rats). Drawings adapted from Paxinos, George; Watson, Charles. (1998). The Rat Brain in Stereotaxic Coordinates. London: Academic Press.

Experiment 1 was conducted in a single group of rats, using a between‐subjects design (three experimental groups with the random allocation of rats into groups using the random number generator function on Windows Excel; sensor placements shown in Fig. 1a). Experiments 2 and 3 were conducted in a separate group of rats, using a within‐subjects design (each rat received each treatment in each experiment (no blinding of experimenter); sensor placements shown in Fig. 1b). A timeline diagram of experimental procedure, including number of animals per single experiment, excluded animals and reason for exclusion is given in Figure S1.

By adopting a within‐subjects design in experiment 2 & 3, the data variability and thus the number of animals required for statistical significance could be reduced. The animals’ suffering during experiments was minimized using a wireless glutamate biosensor technology which allows performing experiments in awake, freely moving rats. Adequate anaesthesia during surgery and pain medication, as outlined above, together with close monitoring of the animals after the surgery ensured reducing the animals’ pain or discomfort during experiments.

### Experiment 1: Effect of NMDA receptor antagonism on mPFC glutamate efflux

A single group of rats (*n* = 4–6 per treatment group) received saline (s.c.) followed 1 h later by either saline (*n* = 2; individual data shown), 0.03 mg/kg (*n* = 4) or 0.06 mg/kg (*n* = 6) MK801 (s.c.). ‘Time’ and ‘drug’ (vehicle, MK801) served as within‐subject factors, whereas ‘dose’ (0, 0.03, 0.06 mg/kg) served as a between‐subjects factor. A vehicle‐vehicle group was used as an additional control to assess the stability of the glutamate‐related signal following repeated injections.

### Experiment 2: Effects of positive and negative mGluR5 allosteric modulation on mPFC glutamate efflux

The effects of ADX47273 and RO4917523 on extracellular glutamate levels in the mPFC were evaluated. ‘Time’ and ‘drug’ served as within‐subject factors. Rats were administered vehicle (10% Tween 80 (0.1% v/v) and 90% natrosol (0.5% v/v); p.o) followed 3 h later by ADX47273 (100 mg/kg, p.o.; *n* = 10) or RO4917523 (0.3 mg/kg, p.o.; *n* = 8).

### Experiment 3: Interactive effects of NMDA receptor antagonism and positive allosteric mGluR5 modulation on mPFC glutamate efflux

The interactive effects of MK801 (0.03 mg/kg) on mPFC extracellular glutamate efflux and ADX47273 pre‐treatment were assessed. ‘Time’ and ‘drug’ served as within‐subject factors. Rats were administered saline (s.c.) *or* vehicle (10% Tween 80 (0.1% v/v) and 90% natrosol (0.5% v/v); p.o.) followed in turn by MK801 (s.c.) *or* ADX47273 (p.o.) followed by MK801 (s.c.) in a counter‐balanced fashion (*n* = 10). ADX47273 was administered before MK801 to ensure appropriate drug exposures were achieved. ADX47273 has a *T*_max_ of 1.5 h, whereas MK801 has a *T*_max_ of 15 min. Thus, the time points at which glutamate efflux was measured, maximal exposures of ADX47273 and MK801 were attained. As an additional control, rats were subsequently administered ADX47273 (p.o.) followed by saline (s.c.). We did not assess the effects of RO4917523 on MK801‐induced glutamate efflux since RO4917523 failed to modulate the disruptive effects of MK801 on behavioural performance on the 5‐CSRTT (Isherwood *et al*. 2015).

### Histology

At the end of the experiments, rats were anaesthetized with a lethal dose of sodium pentobarbital (0.16 mg/kg, 2 mL/kg; Merial, Germany) and perfused transcardially at a rate of 10 mL/min with PBS (Sigma‐Aldrich, Germany) followed by 4% paraformaldehyde (PFA, Alfa Aesar^®^, Germany). The brains were carefully removed, post‐fixed in 4% PFA at ca. 22°C for 24 h and subsequently transferred to 30% sucrose for cryo‐protection for 24 h. The brains were then removed and frozen at −20°C until sectioned. Coronal sections, cut at 50 μm (LEICA cm3050, Biosystems, Germany) were thawed onto Superfrost Ultra Plus^®^ microscope slides (Thermo Scientific, Germany) and frozen at −20°C until placement assessment. Brain sections were defrosted, dried and subsequently incubated in 4% PFA for 5 min, before being allowed to dry overnight before analysis of probe placement.

### Data acquisition and statistical analysis

Data were acquired using Serenia^®^ acquisition software (Pinnacle Technology Inc.) and analysed using Spike2 (Version 8; Cambridge Electronic Design Limited, UK). Glutamate levels were recorded every second for 2–3 h following drug injection. Data were averaged into 10 min time bins. Raw data (nA) were converted to glutamate concentration (nM) based on the calibration curve generated for each sensor during the calibration procedure. The relative change in glutamate concentration (nM) is reported (i.e. raw data minus baseline). Sample sizes were predetermined based on previous studies and verified statistically by a non‐clinical biostatistician using *Proc Power* in SAS Version 9.2 (SAS Institute Inc., Cary, NC, USA). Primary parameter for sample size calculation was drug‐induced ‘change in glutamate release’ versus vehicle treatment (power of 80% to determine drug‐induced change in glutamate release of at least 50% vs. control group with *t*‐test, assuming heteroscedastic variance of α = 5%).

All statistical analyses were conducted using SPSS for Windows (version 21) and GraphPad Prism 7.02. Statistical significance was set at *p *<* *0.05. Three animals that lost head caps in the course of the experiment were excluded from further analysis as outlined in detail in the results. No test of outliers was carried out. In all studies, data were analysed by repeated‐measures anova. In the study involving MK801 alone, treatment and time served as within‐ and between‐subject factors, respectively (mixed design anova). In all other studies, treatment and time served as within‐subject factors. When significant main effects and interactions were found, further analysis using Bonferroni *post hoc* tests were performed. No additional test of normality was performed. For data represented in the form of a histogram, positive and negative changes in glutamate are represented additively, with the baseline set as zero.

## Results

### Drug bioavailability

Plasma levels of MK801, ADX47273 and RO4917523 are shown in Table 1. MK801 levels 15 min after administration were comparable to previously published levels in Lister‐hooded rats (Isherwood *et al*. 2015), specifically 7.64 nM in Wistar Han rats compared to 6.74 nM in Lister‐hooded rats. Plasma levels of ADX47273 and RO4917523 were also broadly similar to those previously reported (Isherwood *et al*. 2015). However, ADX47273 achieved moderately lower plasma exposures in Wister Han rats compared with Lister‐hooded rats, whereas RO4917523 achieved moderately higher exposures in this rat strain. However, it should be noted that blood samples were collected 15 min before those taken from the Lister‐hooded rats. Nevertheless, plasma exposures in Wistar Han rats were sufficient to evoke a pharmacological response, based on functional *in vitro* data reported previously (Isherwood *et al*. 2015) and this study.

### Histology

Reconstructed active zones of the glutamate biosensors (1 mm) are shown in Fig. 1a and b. Two rats were excluded from experiment 1 (MK801 dose–response) due to misaligned placements. Sensors were predominately located in the prelimbic and infralimbic subregions of the mPFC.

### Experiment 1: Effect of NMDA receptor antagonism on mPFC glutamate efflux

**Figure 2:**
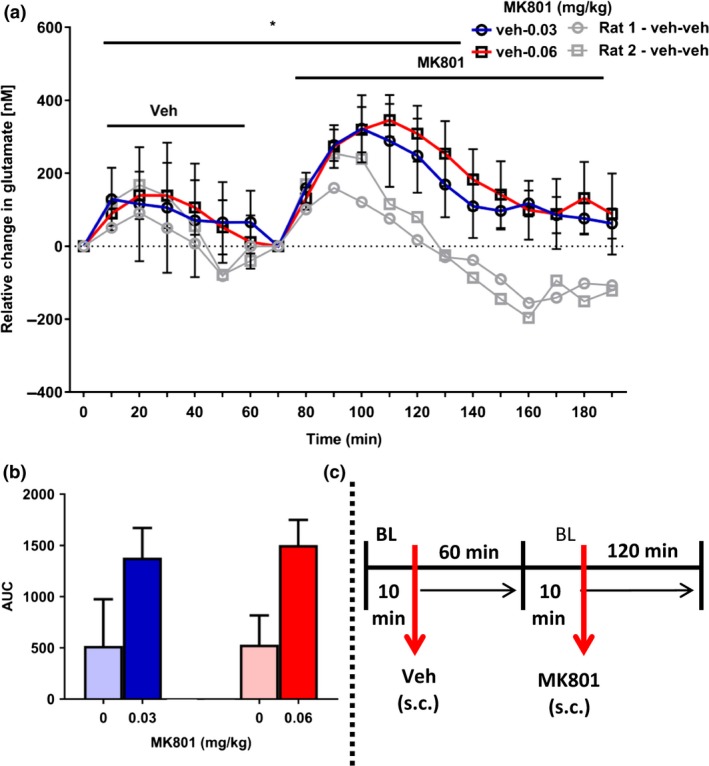
Effect of NMDA receptor antagonism, by 0.03 or 0.06 mg/kg MK801 (s.c.), on extracellular glutamate efflux in the mPFC. (a) All rats received vehicle (*n* = 12 rats; s.c.; time bin zero), followed by vehicle (grey) (*n* = 2 rats; individual data shown) or MK801 (0.03 mg/kg (*n* = 4 rats) or 0.06 mg/kg (*n* = 6 rats) 1 h later. Data points represent mean ± 1SEM. Repeated‐measures anova (mixed design) (main effect of drug, *p <* *0.05 vehicle versus MK801 (up to 1 h post‐injection). (b) area under the curve (AUC) presented, as a histogram, for vehicle and MK801 treatment, 1 h post‐injection. (c) Summary of experimental timeline (BL = baseline).

Glutamate‐related signals were stable approximately 1 h after the start of the experiment, at which point a 10 min baseline period was recorded. The effects of a single injection of saline (s.c.) on glutamate efflux was then monitored for 1 h. Consistent with a previous report (Moghaddam *et al*. 1997), the injection procedure produced an immediate but modest increase in glutamate efflux (Fig. 2a). One hour later, a new baseline was recorded, after which saline, 0.03 or 0.06 mg/kg MK801 (s.c.) was administered. Unfortunately, for technical reasons, two saline control animals were excluded from further analysis. Specifically, during the experiment the lithium batteries leaked inside the head caps, shorting the internal electrical circuit on the potentionstat and preventing the glutamate signal being recorded. MK801 produced a significant, approximate 2‐fold increase in glutamate efflux (main effect of drug, *F*_1,8_ = 6.9, *p *<* *0.05) compared with the saline control (Fig. 2a); an effect that did not depend on the dose of MK801 administered (main effect of dose; drug × dose interaction, *NS*). The increase in glutamate concentration following 0.03 and 0.06 mg/kg MK801 was apparent in the first 10 min time bin and peaked after 30 min at 322 ± 92 nM following the 0.03 mg/kg dose, and at 345 ± 45 nM, 40 min after the 0.06 mg/kg dose (Fig. 2a). We also observed an apparent increase in glutamate efflux following the vehicle injection. Within 2 h of MK801 administration, the extracellular concentration of glutamate in the mPFC returned to baseline. Despite modest differences in glutamate response between 0.03 and 0.06 mg/kg MK801, there was no overall difference in response, as revealed by the area under the curve (AUC) for the vehicle and MK801 groups [AUC represents 1 h post‐vehicle/MK801 administration (i.e. time bin 0 to bin 8)] (Fig. 2b).

### Experiment 2: Effect of allosteric mGluR5 modulation on mPFC glutamate efflux

**Figure 3:**
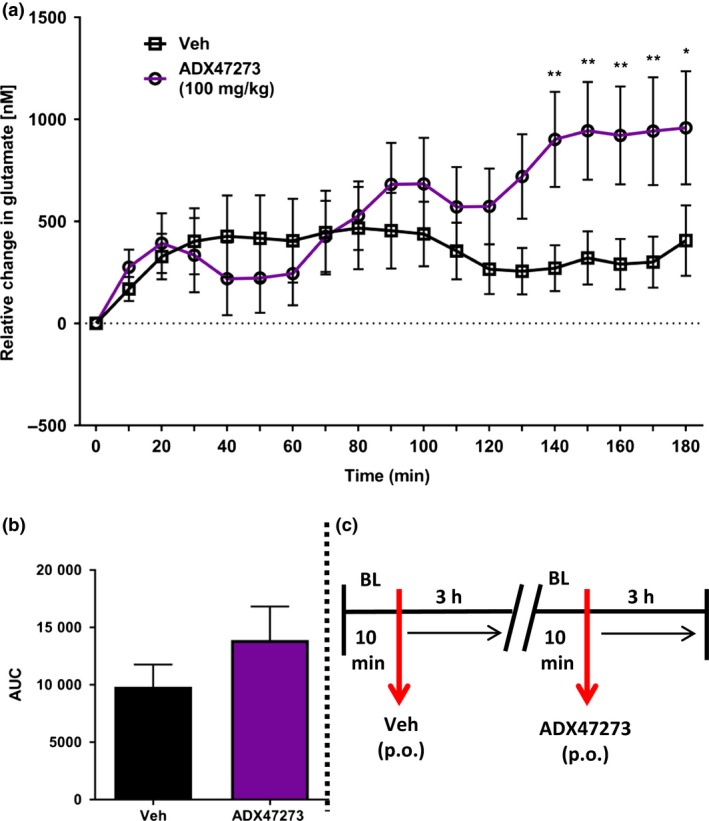
Effect of the positive allosteric mGluR5 modulator, S‐(4‐Fluoro‐phenyl)‐{3‐[3‐(4‐fluoro‐phenyl)‐[1,2,4]‐oxadiazol‐5‐yl]‐piperidin‐1‐yl}‐methanone (ADX47273), on extracellular glutamate efflux in the mPFC (*n* = 10 rats). (a) All rats received vehicle (p.o.; time bin zero). Three hours later, all rats received ADX47273 (100 mg/kg, p.o.; time bin zero). Data points represent mean ± 1 SEM. Repeated‐measures anova, Bonferroni *post hoc*, *p <* *0.05, **p <* *0.01, ADX47273 versus vehicle treatment. (b) area under the curve (AUC) presented, as a histogram, for vehicle and ADX47273 conditions. (c) Summary of experimental timeline (BL = baseline).

The effect the positive allosteric mGluR5 modulator, ADX47273, on glutamate efflux in the mPFC is shown in Fig. 3. One rat lost his head cap and was removed from this experiment. Glutamate‐related signals were stable after approximately 1 h, whereupon a 10 min baseline was recorded. All rats then received vehicle (p.o.) followed by a further 3 h of recording. The administration of vehicle evoked an immediate, but relatively small increase in glutamate efflux (~325–400 nM), which was maintained for approximately 2 h. All rats were then administered ADX47273 (p.o.) following an additional 10 min re‐baseline and glutamate efflux recorded for a further 3 h. ADX47273 produced a significant but delayed increase in glutamate efflux within the mPFC compared with the vehicle control group; (drug × time interaction: *F*_18,162_ = 3.4, *p *<* *0.001; main effect of drug, *NS*) (Fig. 3a). *Post hoc* tests revealed that ADX47273 significantly increased glutamate efflux relative to the vehicle group 140 min after its systemic administration. At this time, glutamate efflux was approximately 3‐fold higher than the vehicle group (vehicle, 271 ± 112 nM vs. ADX47273, 902 ± 233 nM, *p *<* *0.01). AUC data for the control and ADX47273 groups, collapsed over 3 h (i.e. from time bin 0), are shown in Fig. 3b.

**Figure 4:**
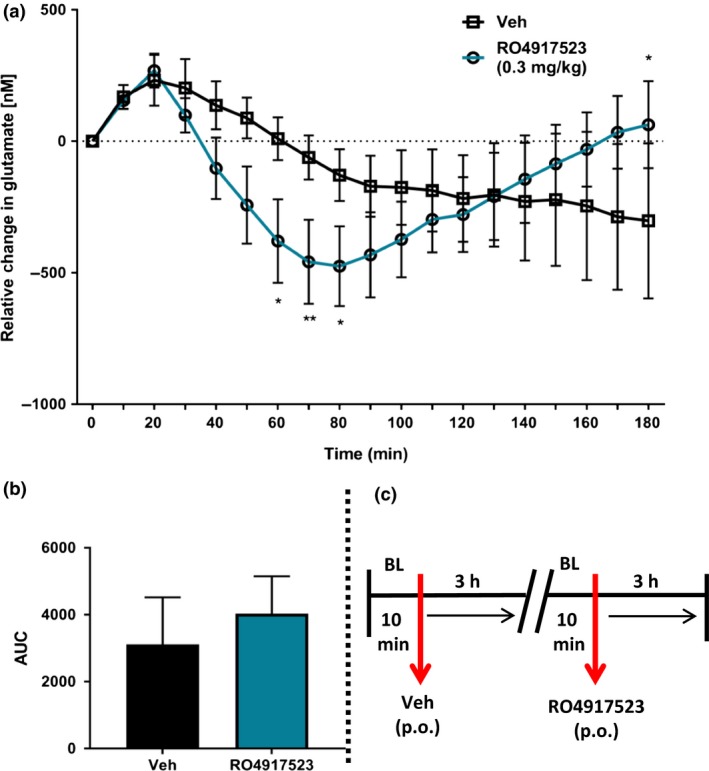
Effect of the negative allosteric mGluR5 modulator, RO4917523, on extracellular glutamate efflux in the mPFC (*n* = 8 rats). (a) All rats received vehicle (p.o.; time bin zero). Three hours later, rats received RO4917523 (0.3 mg/kg, p.o.; time bin zero). Data points represent mean ± 1SEM. Repeated‐measures anova, Bonferroni *post hoc*, *p <* *0.05, **p <* *0.01, RO4917523 versus vehicle condition. (b) area under the curve (AUC) presented, as a histogram, for vehicle and RO4917523 conditions. (c) Summary of experimental timeline (BL = baseline).

Owing to lost head caps, two rats were excluded from the RO4917523 study. One additional rat was excluded from this group because of an unstable baseline, possibly caused by sensor damage during implantation. Glutamate‐related signals were stable after 1 h. Following a 10 min baseline, rats were then administered vehicle (p.o.) followed by 3 h of recording. Again, vehicle administration caused an immediate but small increase in glutamate efflux (~230 nM). This effect peaked after 20 min and returned to baseline levels within 70 min (Fig. 4a). For the remainder of the experiment, glutamate efflux declined, eventually reaching levels approximately 300 nM below baseline. RO4917523 significantly decreased glutamate efflux (drug × time interaction: *F*_18,126_ = 4.3, *p *<* *0.001; main effect of drug, *NS*), reaching statistical significance 60 min after its administration compared with the vehicle group. A maximal decrease in glutamate efflux of approximately observed after 80 min (vehicle, 129 ± 99 nM decrease vs. RO4917523, 475 ± 152nM decrease, *p *<* *0.05). A gradual increase in glutamate efflux was then observed, reaching levels that were largely indifferent to that measured at baseline suggesting a return of glutamatergic tone in the mPFC. AUC data for the control and RO4917523 groups, collapsed over 3 h (i.e. from time bin 0), are shown in Fig. 4b.

### Experiment 3: Combined effects of NMDA receptor antagonism and positive allosteric mGluR5 modulation on mPFC glutamate efflux

**Figure 5:**
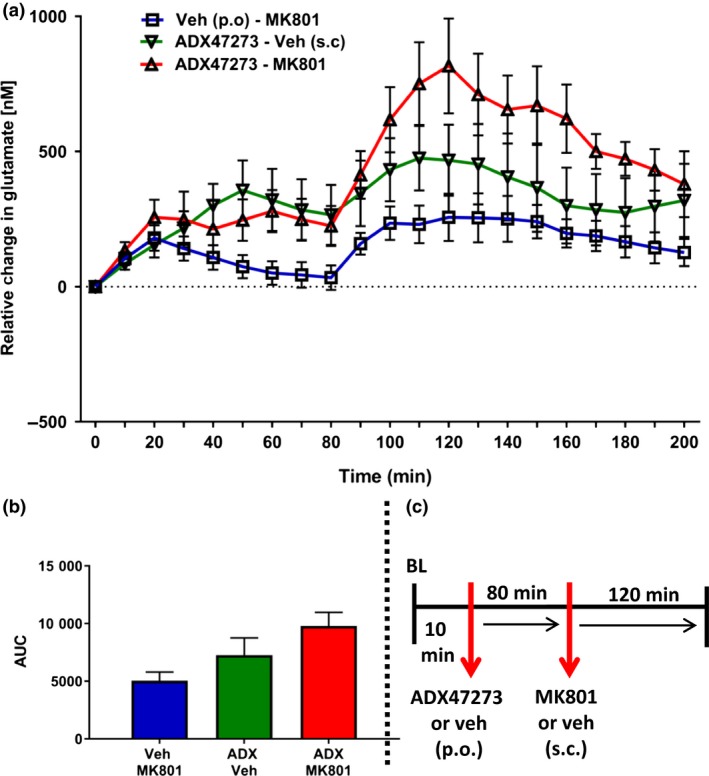
Effect of pre‐treatment with S‐(4‐Fluoro‐phenyl)‐{3‐[3‐(4‐fluoro‐phenyl)‐[1,2,4]‐oxadiazol‐5‐yl]‐piperidin‐1‐yl}‐methanone (ADX47273) on glutamate efflux in the mPFC evoked by the NMDA receptor antagonist MK801 (*n* = 10 rats). (a) Rats received vehicle (p.o.) or ADX47273 (100 mg/kg, p.o.; time bin zero), followed by vehicle (s.c.) or MK801 (0.03, s.c.) 80 min later. Data points represent mean ±1SEM. Repeated‐measures anova. (b) area under the curve (AUC) presented as a histogram, for vehicle or ADX47273 or MK801 or ADX47273 and MK801 treatment (2‐h post‐MK801/vehicle injection). (c) Summary of experimental timeline (BL = baseline).

One rat was excluded from this study because of a lost head cap. Fig. 5 shows the effect of pre‐treatment with ADX47273 on glutamate efflux evoked by MK801; vehicle data are shown in Fig. 2. Within 1 h of habituation, glutamate‐related signals were stable and recording commenced. After a 10 min baseline, rats received either vehicle (p.o.) or ADX47273 (100 mg/kg, p.o), which evoked an immediate, but relatively small, increase in glutamate efflux. Eighty minutes after ADX47273/vehicle treatment, rats received MK801 (0.03 mg/kg, s.c.) or vehicle (s.c.) with glutamate efflux recorded for an additional 2 h (Fig. 5a; Fig. S2). Overall, anova revealed a significant drug x time interaction (*F*_24,216_ = 3.5, *p *<* *0.001) and consistent with previous experiments glutamate levels declined steadily in the vehicle control group. Glutamate efflux increased maximally in ADX47273‐treated and MK801‐treated rats by 476 ± 119 nM and 257 ± 88 nM, respectively (Fig. 5a). However, when combined, ADX7273 and MK801 increased glutamate efflux by 817 ± 176 nM. AUC data for the various groups, over 2 h (i.e. from time bin 8), are shown in Fig. 5b.

## Discussion

We investigated the effects of allosteric mGluR5 modulation on prefrontal glutamate efflux and specifically whether the cognitive enhancing effects of positive mGluR5 modulation is reflected by a reversal of increased glutamate efflux induced by NMDA receptor antagonism. In this context numerous studies have demonstrated that NMDA receptor antagonists increase glutamate efflux and disrupt cognitive functions dependent on the PFC (Moghaddam *et al*. 1997; Moghaddam and Adams 1998; Campbell *et al*. 2004; Ceglia *et al*. 2004; Homayoun *et al*. 2004; Lopez‐Gil *et al*. 2007; Stefani and Moghaddam 2010; Fletcher *et al*. 2011; Agnoli and Carli 2012).

In contrast, positive allosteric mGluR5 modulators are hypothesized to offset the disinhibiting effects of NMDA receptor antagonists by augmenting glutamatergic neurotransmission at post‐synaptic NMDA receptors and/or by activating GABA‐ergic interneurons (Shigemoto *et al*. 1993; Romano *et al*. 1995; Kerner *et al*. 1997; Alagarsamy *et al*. 2002; Muly *et al*. 2003; Lecourtier *et al*. 2007). *In vivo*, positive allosteric mGluR5 modulation blocks the effects of MK801 on neuronal firing in the orbitofrontal cortex and mPFC (Lecourtier *et al*. 2007; Homayoun and Moghaddam 2008) whereas negative mGluR5 modulation has the opposite effect (Homayoun and Moghaddam 2006). Therefore, we hypothesized that positive allosteric mGluR5 modulation by ADX47273 would decrease glutamate release by enhancing GABA‐ergic inhibition of glutamatergic pyramidal neurons in the mPFC. Our findings, however, indicate that the positive allosteric mGluR5 modulator ADX47273 increases glutamate efflux in the mPFC when administered alone or when combined with the NMDA receptor antagonist MK801. Moreover, glutamate efflux in the mPFC was decreased by the negative allosteric mGluR5 modulator RO4917523. These findings indicate that cognitive enhancing effects of positive allosteric mGluR5 modulators in tasks that depend on NMDA receptor function may not be because of an attenuation of glutamate release in the PFC.

We previously reported that MK801 increases impulsivity in the 5‐CSRTT (Isherwood *et al*. 2015); an effect hypothesized to originate from increased glutamatergic transmission in the mPFC, specifically within the infralimbic cortex (Moghaddam *et al*. 1997; Moghaddam and Adams 1998; Higgins *et al*. 2003b; Carli *et al*. 2004; Ceglia *et al*. 2004; Murphy *et al*. 2005; Fletcher *et al*. 2011; Pozzi *et al*. 2011). Indeed, ketamine and other NMDA receptor antagonists increase glutamatergic neuronal activity (Suzuki *et al*. 2002; Jodo *et al*. 2003; Jackson *et al*. 2004; Homayoun *et al*. 2005) and consequently extracellular glutamate levels in the mPFC (Moghaddam *et al*. 1997; Adams and Moghaddam 1998; Ceglia *et al*. 2004; Lopez‐Gil *et al*. 2007). Increased availability of glutamate at non‐NMDA receptors is thought to mediate cognitive impairment and impaired response inhibitory control produced by NMDA receptor antagonists (Moghaddam *et al*. 1997; Pozzi *et al*. 2011). Indeed, non‐NMDA receptor antagonists and drugs that suppress glutamate release in the mPFC show efficacy in alleviating behavioural deficits evoked by NMDA receptor antagonism (Bubser *et al*. 1995; Moghaddam *et al*. 1997; Moghaddam and Adams 1998; Pozzi *et al*. 2011). For example mGluR2/3 agonists block NMDA receptor antagonist‐induced glutamate efflux, primarily by decreasing neuronal firing in the mPFC (Moghaddam and Adams 1998; Homayoun *et al*. 2005; Pozzi *et al*. 2011), whilst also attenuating impulsivity in the 5‐CSRTT, locomotion, stereotypy and deficits in working memory (Moghaddam and Adams 1998; Homayoun *et al*. 2005; Pozzi *et al*. 2011).

The paradoxical effect of NMDA receptor antagonists to increase glutamate release within the mPFC is hypothesized to result from the inhibition of GABA‐ergic interneurons and thus the disinhibition of glutamatergic pyramidal neurons (Moghaddam *et al*. 1997; Krystal *et al*. 2003; Homayoun and Moghaddam 2007). However, although local infusion of 3‐(2‐carboxypiperazin‐4‐yl)propyl‐1‐phosphonic acid (CPP), a competitive NMDA receptor antagonist, increases mPFC glutamate release (Ceglia *et al*. 2004; Pozzi *et al*. 2011), MK801 and other non‐competitive NMDA receptor antagonists have no effect on glutamate release (Suzuki *et al*. 2002; Lorrain *et al*. 2003; Lopez‐Gil *et al*. 2007) or glutamatergic neuronal firing in the mPFC (Suzuki *et al*. 2002; Jodo *et al*. 2005). Thus, the population of NMDA receptors responsible for the effects of MK801 on glutamate release appear to be located outside the PFC, possibly on GABA‐ergic neurons tonically involved in inhibiting glutamatergic input to the PFC (Lopez‐Gil *et al*. 2007).

**Figure 6:**
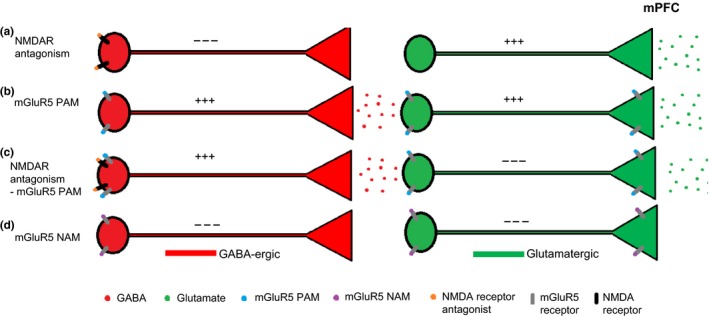
Illustrative diagram showing putative mechanisms underlying the effects of NMDA receptor antagonism and allosteric mGluR5 modulation on glutamate efflux in the mPFC. (a) NMDA receptor antagonism inhibits GABA‐ergic neurons leading to a disinhibition of glutamatergic neurons and increased glutamate efflux. (b) Positive allosteric mGluR5 modulation activates GABA‐ergic neurons and glutamatergic neurons *via* the activation of pre‐ and post‐synaptic mGluR5 resulting in increased glutamate efflux. (c) NMDA receptor antagonism and positive allosteric mGluR5 modulation leads to a reversal of (i) NMDA receptor antagonist‐induced disinhibition; (ii) post‐synaptic mGluR5 activation of glutamatergic neurons; (iii) pre‐synaptic mGluR5 autoreceptor activation, leading to increased glutamate efflux. (d) Negative allosteric mGluR5 modulation inhibits GABA‐ergic neurons and glutamatergic neurons leading to decreased glutamate efflux. This is a hypothesized proposal since we did not assess GABA‐ergic mechanisms.

In this study, ADX47273 increased glutamate efflux in the mPFC when administered alone. This effect is compatible with a previous report describing the excitatory effects of the positive allosteric mGluR5 modulator 3‐cyano‐N‐(1,3‐diphenyl‐1H‐pyrazol‐5‐yl)benzamide (CDPPB) on neuronal firing in the mPFC (Lecourtier *et al*. 2007). Since ADX47273 increased rather than decreased glutamate efflux, our results suggest that excitation of glutamatergic neurons by mGluR5 PAM was sufficient to overcome the assumed enhanced inhibition of these neurons by mGluR5‐induced depolarization of GABA‐ergic neurons, as depicted in Fig. 6. Moreover, the temporal profile and sustained increase in glutamate efflux evoked by ADX47273 we report mirrors the bioavailability of this compound in plasma after oral dosing (Isherwood *et al*. 2015). Of particular importance, our findings support the conclusion that allosteric modulators do not lead to a rapid desensitization of the target receptor (Urwyler 2011), unlike orthosteric modulators (Gereau and Heinemann 1998).

Negative allosteric mGluR5 modulation by RO4917523 produced a significant reduction in glutamate efflux in the mPFC, a finding consistent with the effects of the mGluR5 antagonist 2‐Methyl‐6‐(phenylethynyl)pyridine (MPEP), which decreases burst activity and firing of mPFC neurons (Homayoun and Moghaddam 2006). Again, these findings suggest a post‐synaptic site of action of RO4917523 on glutamatergic pyramidal neurons. However, the initial and rapid effect of RO4917523 on glutamate efflux was surprising given the known pharmacokinetic properties of RO4917523 (*T*_max_ 2 h) (Isherwood *et al*. 2015); changes in glutamate efflux occurred *before* optimal plasma exposures were attained (i.e. within 30 min). In the pharmacokinetic study described, a dose of 5.8 mg/kg (p.o.) was used. An additional pharmacokinetic study was therefore performed using a lower dose of RO4917523. Importantly, the *C*_max_ and *T*_max_ of RO4917523 (0.1 mg/kg, p.o.) were determined to be 96 nM and 25 min, respectively, whereas *in vitro,* the IC_50_ of RO4917523 was ~4.5 nM (Isherwood *et al*. 2015). Since the brain/plasma ratio of RO491723 is ~2–3 and the predicted free‐fraction is 3% we can assume that following 0.1 mg/kg RO4917523 administration, an active brain exposure of ~6 nM would be attained. This is well within the range of the *in vitro* IC_50_. In this study, a dose of 0.3 mg/kg was used, suggesting with certainty that appropriate brain exposures were attained within 25 min of administration to evoke a pharmacological effect at mGluR5. Moreover, our analysis also revealed that within 2 h of administration (0.1 mg/kg), the predicted exposure of this compound in the brain would fall below the *in vitro* IC_50_. This would explain the return of glutamatergic tone in the mPFC at this time.

It is important to note that some variability in glutamate release was observed in experiments 1 and 2 following the administration of the vehicle. Thus, in experiment 1, the vehicle injection produced an increase in glutamate efflux of approximately 200 nM, whereas in experiment 2 the vehicle injection increased glutamate release by almost 400 nM. The reason for this variation remains unclear but may be because of different vehicle solutions for experiment 1 (saline) and experiment 2 (10% Tween 80), differences in the dynamic *in vivo* performance of the sets of biosensors used for each experiment, and/or that the biosensors were implanted in a slightly different anterior–posterior location. Thus, whereas probe placements in experiment 1 ranged between 3.2 and 3.7 mm along the anterior–posterior axis, placements in experiment 2 ranged between 2.2 and 3.2 mm. It is therefore possible that the observed variability in glutamate release resulted from differential effects of injection stress on glutamatergic neurons located in posterior and anterior regions of the mPFC.

The main findings of this study indicate that positive allosteric mGluR5 modulation and NMDA receptor antagonism lead the same outcome of increasing glutamate release in the PFC. It is unlikely therefore that the reported ability of mGluR5 PAMs to reverse cognitive impairments induced by NMDA receptor antagonism is related to a correction of glutamate release in this region. Indeed, results from several studies indicate that mGluR5 PAMs (e.g. ADX47273 and CDPPB) ameliorate a range of deficits caused by MK801 and Phenylcyclohexylpiperidine (PCP), including set‐shifting (Stefani and Moghaddam 2010; LaCrosse *et al*. 2015), novel object recognition (Horio *et al*. 2013) and impulsivity on the 5‐choice task (Isherwood *et al*. 2015). Although the neural substrates underlying the cognitive enhancing effects of mGluR5 PAMs are presently unknown these may be mediated in part by effects within the mesolimbic dopamine system. Thus, in addition to stimulating glutamatergic transmission, NMDA receptor blockade increases dopamine efflux in the nucleus accumbens (Adams and Moghaddam 1998, 2001; Moghaddam and Adams 1998; Mathé *et al*. 1999), possibly by stimulating glutamatergic outflow from the PFC to subsequently enhance activity in the mesolimbic dopamine system (Murase *et al*. 1993; Mathé *et al*. 1999). Moreover, systemic ADX47273 decreases dopamine efflux in the nucleus accumbens (Liu *et al*. 2008). However, although this may account for the ability of ADX47273 to reduce MK801‐induced impulsivity in the 5‐choice task (Isherwood *et al*. 2015), for which performance depends on dopaminergic mechanisms in the nucleus accumbens (Robbins 2002), there is at least one report showing that CDPPB does not alter basal or MK801‐induced dopamine release in the nucleus accumbens (Lecourtier *et al*. 2007). Thus, it remains an open question whether the pro‐cognitive effects of ADX47272 are mediated by an attenuation of MK801‐induced release of dopamine and other neurotransmitters (e.g. within in the nucleus accumbens). Fig. 6 proposes a hypothesized mechanism for the modulation of glutamate release within the mPFC by NMDA receptor antagonism and mGluR5 modulation.

In conclusion, our findings demonstrate that positive allosteric mGluR5 modulation by systemic ADX47272 leads to a sustained elevation in extracellular glutamate concentration in the mPFC. Rather than reversing the effects of the MK801 on glutamate efflux in this region, as expected, ADX47272 produced an additive increase in glutamate availability that contrasted with the effects of the negative allosteric mGluR5 modulator RO4917523. Therefore, the cognitive enhancing effects of mGluR5 PAMs in tasks dependent on NMDA receptor function appear to be dissociable from alterations in PFC glutamate release. These findings imply an additional mechanism responsible for the cognitive enhancing effects of mGluR5 PAMs separate from the reversal of the NMDA receptor antagonist‐induced disinhibition of glutamatergic neurons in the PFC (Moghaddam *et al*. 1997; Krystal *et al*. 2003; Homayoun and Moghaddam 2007). Defining the neural loci underlying the pro‐cognitive effects of mGluR5 PAMs may provide new opportunities to ameliorate cognitive dysfunction present in a range of neuropsychiatric disorders including schizophrenia.

## Supporting information

**Figure S1**. Bidirectional variation in glutamate efflux in the medial prefrontal cortex induced by selective positive and negative allosteric mGluR5 modulators. Sarah N. Isherwood, Trevor W. Robbins, Jeffrey W. Dalley, Anton Pekcec.
**Figure S2.** Schematic diagram illustrating the experimental timeline for each experiment; n numbers represent the total number of rats used in each experiment (i.e. before exclusion); exclusion reason included: *TF*, technical fault; *PP*, incorrect probe placement; *HC*, lost head cap.

## References

[jnc14290-bib-0001] Abekawa T. , Honda M. , Ito K. and Koyama T. (2003) Effects of NRA0045, a novel potent antagonist at dopamine D4, 5‐HT2A, and α1 adrenaline receptors, and NRA0160, a selective D4 receptor antagonist, on phencyclidine‐induced behavior and glutamate release in rats. Psychopharmacology 169, 247–256. 12898123 10.1007/s00213-003-1517-8

[jnc14290-bib-0002] Adams B. and Moghaddam B. (1998) Corticolimbic dopamine neurotransmission is temporally dissociated from the cognitive and locomotor effects of phencyclidine. J. Neurosci. 18, 5545–5554. 9651235 10.1523/JNEUROSCI.18-14-05545.1998 PMC6793475

[jnc14290-bib-0003] Adams B. W. and Moghaddam B. (2001) Effect of clozapine, haloperidol, or M100907 on phencyclidine‐activated glutamate efflux in the prefrontal cortex. Biol. Psychiatry 50, 750–757. 11720693 10.1016/s0006-3223(01)01195-7

[jnc14290-bib-0004] Agnoli L. and Carli M. (2012) Dorsal–striatal 5‐HT2A and 5‐HT2C receptors control impulsivity and perseverative responding in the 5‐choice serial reaction Time Task. Psychopharmacology 219, 633–645. 22113450 10.1007/s00213-011-2581-0

[jnc14290-bib-0005] Alagarsamy S. , Rouse S. T. , Junge C. , Hubert G. W. , Gutman D. , Smith Y. and Conn P. J. (2002) NMDA‐induced phosphorylation and regulation of mGluR5. Pharmacol. Biochem. Behav. 73, 299–306. 12117583 10.1016/s0091-3057(02)00826-2

[jnc14290-bib-0006] Amitai N. and Markou A. (2010) Disruption of performance in the five‐choice serial reaction time task induced by administration of N‐methyl‐D‐aspartate receptor antagonists: relevance to cognitive dysfunction in schizophrenia. Schizophr. N‐Methyl–Aspartate Recept. Dysfunct. Cortical Connect. 68, 5–16. 10.1016/j.biopsych.2010.03.004 PMC2900523 20488434

[jnc14290-bib-0007] Amitai N. , Semenova S. and Markou A. (2007) Cognitive‐disruptive effects of the psychotomimetic phencyclidine and attenuation by atypical antipsychotic medications in rats. Psychopharmacology 193, 521–537. 17497138 10.1007/s00213-007-0808-x

[jnc14290-bib-0008] Archer T. and Garcia D. (2016) Attention‐deficit/hyperactivity disorder: focus upon Aberrant N‐Methyl‐D‐aspartate receptors systems. Curr. Top. Behav. Neurosci. 29, 295–311. 26718589 10.1007/7854_2015_415

[jnc14290-bib-0009] Aultman J. M. and Moghaddam B. (2001) Distinct contributions of glutamate and dopamine receptors to temporal aspects of rodent working memory using a clinically relevant task. Psychopharmacology 153, 353–364. 11271408 10.1007/s002130000590

[jnc14290-bib-0010] Barker G. R. I. and Warburton E. C. (2015) Object‐in‐place associative recognition memory depends on glutamate receptor neurotransmission within two defined hippocampal‐cortical circuits: a critical role for AMPA and NMDA receptors in the hippocampus, perirhinal, and prefrontal cortices. Cereb. Cortex N. Y. NY 25, 472–481. 10.1093/cercor/bht245 PMC4380082 24035904

[jnc14290-bib-0011] Bubser M. , Tzschentke T. and Hauber W. (1995) Behavioural and neurochemical interactions of the AMPA antagonist GYKI 52466 and the non‐competitive NMDA antagonist dizocilpine in rats. J. Neural Transm. Gen. Sect. 101, 115–126. 8695042 10.1007/BF01271550

[jnc14290-bib-0012] Campbell U. C. , Lalwani K. , Hernandez L. , Kinney G. G. , Conn P. J. and Bristow L. J. (2004) The mGluR5 antagonist 2‐methyl‐6‐(phenylethynyl)‐pyridine (MPEP) potentiates PCP‐induced cognitive deficits in rats. Psychopharmacology 175, 310–318. 15024550 10.1007/s00213-004-1827-5

[jnc14290-bib-0013] Carli M. , Baviera M. , Invernizzi R. and Balducci C. (2004) The serotonin 5‐HT2A receptors antagonist M100907 prevents impairment in attentional performance by NMDA receptor blockade in the rat prefrontal cortex. Neuropsychopharmacology 29. 10.1038/sj.npp.1300479 15127084

[jnc14290-bib-0014] Castner S. A. and Williams G. V. (2007) Tuning the engine of cognition: A focus on NMDA/D1 receptor interactions in prefrontal cortex. Brain Cogn. 63, 94–122. 17204357 10.1016/j.bandc.2006.11.002

[jnc14290-bib-0015] Ceglia I. , Carli M. , Baviera M. , Renoldi G. , Calcagno E. and Invernizzi R. W. (2004) The 5‐HT2A receptor antagonist M100,907 prevents extracellular glutamate rising in response to NMDA receptor blockade in the mPFC. J. Neurochem. 91, 189–199. 15379899 10.1111/j.1471-4159.2004.02704.x

[jnc14290-bib-0016] Chu Z. and Hablitz J. J. (1998) Activation of group I mGluRs increases spontaneous IPSC frequency in rat frontal cortex. J. Neurophysiol. 80, 621–627. 9705455 10.1152/jn.1998.80.2.621

[jnc14290-bib-0017] Deakin J. F. W. , Slater P. , Simpson M. D. C. , Gilchrist A. C. , Skan W. J. , Royston M. C. , Reynolds G. P. and Cross A. J. (1989) Frontal cortical and left temporal glutamatergic dysfunction in schizophrenia. J. Neurochem. 52, 1781–1786. 2566649 10.1111/j.1471-4159.1989.tb07257.x

[jnc14290-bib-0018] Driesen N. R. , McCarthy G. , Bhagwagar Z. *et al* (2013) The impact of NMDA receptor blockade on human working memory‐related prefrontal function and connectivity. Neuropsychopharmacology 38, 2613–2622. 23856634 10.1038/npp.2013.170 PMC3828532

[jnc14290-bib-0019] Fletcher P. J. , Rizos Z. , Noble K. and Higgins G. A. (2011) Impulsive action induced by amphetamine, cocaine and MK801 is reduced by 5‐HT2C receptor stimulation and 5‐HT2A receptor blockade. Serotonin New Wave 61, 468–477. 10.1016/j.neuropharm.2011.02.025 21402085

[jnc14290-bib-0020] Gereau R. W. and Heinemann S. F. (1998) Role of protein kinase C phosphorylation in rapid desensitization of metabotropic glutamate receptor 5. Neuron 20, 143–151. 9459450 10.1016/s0896-6273(00)80442-0

[jnc14290-bib-0021] Goff D. C. and Coyle J. T. (2001) The emerging role of glutamate in the pathophysiology and treatment of schizophrenia. Am. J. Psychiatry 158, 1367–1377. 11532718 10.1176/appi.ajp.158.9.1367

[jnc14290-bib-0022] Graybeal C. , Kiselycznyk C. and Holmes A. (2012) Stress‐induced impairments in prefrontal‐mediated behaviors and the role of the N‐methyl‐D‐aspartate receptor. Neuroscience 211, 28–38. 22414923 10.1016/j.neuroscience.2012.02.042 PMC3351527

[jnc14290-bib-0023] Higgins G. A. , Enderlin M. , Haman M. and Fletcher P.J. , (2003b). The 5‐HT2A receptor antagonist M100,907 attenuates motor and “impulsive‐type” behaviours produced by NMDA receptor antagonism. Psychopharmacology (Berl.) 170, 309–319. 12904968 10.1007/s00213-003-1549-0

[jnc14290-bib-0024] Homayoun H. and Moghaddam B. (2006) Bursting of prefrontal cortex neurons in awake rats is regulated by metabotropic glutamate 5 (mGlu5) receptors: rate‐dependent influence and interaction with NMDA receptors. Cereb. Cortex N. Y. N 1991(16), 93–105. 10.1093/cercor/bhi087 15843630

[jnc14290-bib-0025] Homayoun H. and Moghaddam B. (2007) NMDA receptor hypofunction produces opposite effects on prefrontal cortex interneurons and pyramidal neurons. J. Neurosci. 27, 11496–11500. 17959792 10.1523/JNEUROSCI.2213-07.2007 PMC2954603

[jnc14290-bib-0026] Homayoun H. and Moghaddam B. (2008) Orbitofrontal cortex neurons as a common target for classic and glutamatergic antipsychotic drugs. Proc. Natl Acad. Sci. USA 105, 18041–18046. 19004793 10.1073/pnas.0806669105 PMC2584724

[jnc14290-bib-0027] Homayoun H. , Stefani M. R. , Adams B. W. , Tamagan G. D. and Moghaddam B. (2004) Functional interaction between NMDA and mGlu5 receptors: effects on working memory, instrumental learning, motor behaviors, and dopamine release. Neuropsychopharmacology 29, 1259–1269. 15010696 10.1038/sj.npp.1300417

[jnc14290-bib-0028] Homayoun H. , Jackson M. E. and Moghaddam B. (2005) Activation of metabotropic glutamate 2/3 receptors reverses the effects of NMDA receptor hypofunction on prefrontal cortex unit activity in awake rats. J. Neurophysiol. 93, 1989–2001. 15590730 10.1152/jn.00875.2004

[jnc14290-bib-0029] Horio M. , Fujita Y. and Hashimoto K. (2013) Therapeutic effects of metabotropic glutamate receptor 5 positive allosteric modulator CDPPB on phencyclidine‐induced cognitive deficits in mice. Fundam. Clin. Pharmacol. 27, 483–488. 22594375 10.1111/j.1472-8206.2012.01045.x

[jnc14290-bib-0030] Isherwood S. N. , Pekcec A. , Nicholson J. R. , Robbins T. W. and Dalley J. W. (2015) Dissociable effects of mGluR5 allosteric modulation on distinct forms of impulsivity in rats: interaction with NMDA receptor antagonism. Psychopharmacology 232, 3327–3344. 26063678 10.1007/s00213-015-3984-0

[jnc14290-bib-0031] Jackson M. E. , Homayoun H. and Moghaddam B. (2004) NMDA receptor hypofunction produces concomitant firing rate potentiation and burst activity reduction in the prefrontal cortex. Proc. Natl Acad. Sci. USA 101, 8467–8472. 15159546 10.1073/pnas.0308455101 PMC420417

[jnc14290-bib-0032] Jodo E. , Suzuki Y. , Takeuchi S. , Niwa S. and Kayama Y. (2003) Different effects of phencyclidine and methamphetamine on firing activity of medial prefrontal cortex neurons in freely moving rats. Brain Res. 962, 226–231. 12543474 10.1016/s0006-8993(02)03967-7

[jnc14290-bib-0033] Jodo E. , Suzuki Y. , Katayama T. , Hoshino K.‐Y. , Takeuchi S. , Niwa S.‐I. and Kayama Y. (2005) Activation of medial prefrontal cortex by phencyclidine is mediated via a hippocampo‐prefrontal pathway. Cereb. Cortex N. Y. N 1991(15), 663–669. 10.1093/cercor/bhh168 15342431

[jnc14290-bib-0034] Kalivas P. W. and Volkow N. D. (2011) New medications for drug addiction hiding in glutamatergic neuroplasticity. Mol. Psychiatry 16, 974–986. 21519339 10.1038/mp.2011.46 PMC3192324

[jnc14290-bib-0035] Karlsgodt K. H. , Robleto K. , Trantham‐Davidson H. , Jairl C. , Cannon T. D. , Lavin A. and Jentsch J. D. (2011) Reduced dysbindin expression mediates N‐methyl‐D‐aspartate receptor hypofunction and impaired working memory performance. Biol. Psychiatry 69, 28–34. 21035792 10.1016/j.biopsych.2010.09.012 PMC4204919

[jnc14290-bib-0036] Kerner J. A. , Standaert D. G. , Penney J. B., Jr , Young A. B. and Landwehrmeyer G. B. (1997) Expression of group one metabotropic glutamate receptor subunit mRNAs in neurochemically identified neurons in the rat neostriatum, neocortex, and hippocampus. Mol. Brain Res. 48, 259–269. 9332723 10.1016/s0169-328x(97)00102-2

[jnc14290-bib-0037] Konradi C. and Heckers S. (2003) Molecular aspects of glutamate dysregulation: implications for schizophrenia and its treatment. Pharmacol. Ther. 97, 153–179. 12559388 10.1016/s0163-7258(02)00328-5 PMC4203361

[jnc14290-bib-0038] Krystal J. H. , D'Souza D. C. , Mathalon D. , Perry E. , Belger A. and Hoffman R. (2003) NMDA receptor antagonist effects, cortical glutamatergic function, and schizophrenia: toward a paradigm shift in medication development. Psychopharmacology 169, 215–233. 12955285 10.1007/s00213-003-1582-z

[jnc14290-bib-0039] LaCrosse A. L. , Taylor S. B. , Nemirovsky N. E. , Gass J. T. and Olive M. F. (2015) mGluR5 positive and negative allosteric modulators differentially affect Dendritic spine density and morphology in the prefrontal cortex. CNS Neurol. Disord. Drug Targets 14, 476–485. 25921744 10.2174/1871527314666150429112849 PMC4507801

[jnc14290-bib-0040] Lecourtier L. , Homayoun H. , Tamagnan G. and Moghaddam B. (2007) Positive allosteric modulation of metabotropic glutamate 5 (mGlu5) receptors reverses N‐Methyl‐D‐aspartate antagonist‐induced alteration of neuronal firing in prefrontal cortex. mol. Mech. Brain Dev. Nov. Treat. Mech. Schizophr. 62, 739–746. 10.1016/j.biopsych.2006.12.003 PMC2910402 17511968

[jnc14290-bib-0041] Lindemann L. , Porter R. H. , Scharf S. H. *et al* (2015) Pharmacology of basimglurant (RO4917523, RG7090), a unique metabotropic glutamate receptor 5 negative allosteric modulator in clinical development for depression. J. Pharmacol. Exp. Ther. 353, 213–233. 25665805 10.1124/jpet.114.222463

[jnc14290-bib-0042] Lindsley C. W. , Shipe W. D. , Wolkenberg S. E. , Theberge C. R. , Williams D. L., Jr , Sur C. and Kinney G. G. (2006) Progress towards validating the NMDA receptor hypofunction hypothesis of schizophrenia. Curr. Top. Med. Chem. 6, 771–785. 16719816 10.2174/156802606777057599

[jnc14290-bib-0043] Liu F. , Grauer S. , Kelley C. *et al* (2008) ADX47273 [S‐(4‐Fluoro‐phenyl)‐{3‐[3‐(4‐fluoro‐phenyl)‐[1,2,4]‐oxadiazol‐5‐yl]‐piperidin‐1‐yl}‐methanone]: a Novel Metabotropic Glutamate Receptor 5‐Selective Positive Allosteric Modulator with Preclinical Antipsychotic‐Like and Procognitive Activities. J. Pharmacol. Exp. Ther. 327, 827–839. 18753411 10.1124/jpet.108.136580

[jnc14290-bib-0044] Lopez‐Gil X. , Babot Z. , Amargos‐Bosch M. , Sunol C. , Artigas F. and Adell A. (2007) Clozapine and haloperidol differently suppress the MK‐801‐increased glutamatergic and serotonergic transmission in the medial prefrontal cortex of the rat. Neuropsychopharmacology 32, 2087–2097. 17356574 10.1038/sj.npp.1301356

[jnc14290-bib-0045] Lorrain D. , Baccei C. , Bristow L. , Anderson J. and Varney M. (2003) Effects of ketamine and n‐methyl‐d‐aspartate on glutamate and dopamine release in the rat prefrontal cortex: modulation by a group II selective metabotropic glutamate receptor agonist LY379268. Neuroscience 117, 697–706. 12617973 10.1016/s0306-4522(02)00652-8

[jnc14290-bib-0046] Malhotra A. K. , Pinals D. A. , Weingartner H. , Sirocco K. , Missar C. D. , Pickar D. and Breier A. (1996) NMDA receptor function and human cognition: the effects of ketamine in healthy volunteers. Neuropsychopharmacol. Off. Publ. Am. Coll. Neuropsychopharmacol. 14, 301–307. 10.1016/0893-133X(95)00137-3 8703299

[jnc14290-bib-0047] Mathé J. M. , Nomikos G. G. , Hygge Blakeman K. and Svensson T. H. (1999) Differential actions of dizocilpine (MK‐801) on the mesolimbic and mesocortical dopamine systems: role of neuronal activity. Neuropharmacology 38, 121–128. 10193903 10.1016/s0028-3908(98)00163-4

[jnc14290-bib-0048] Moghaddam B. and Adams B. W. (1998) Reversal of phencyclidine effects by a group II metabotropic glutamate receptor agonist in rats. Science 281, 1349–1352. 9721099 10.1126/science.281.5381.1349

[jnc14290-bib-0049] Moghaddam B. , Adams B. , Verma A. and Daly D. (1997) Activation of glutamatergic neurotransmission by ketamine: a novel step in the pathway from NMDA receptor blockade to dopaminergic and cognitive disruptions associated with the prefrontal cortex. J. Neurosci. 17, 2921–2927. 9092613 10.1523/JNEUROSCI.17-08-02921.1997 PMC6573099

[jnc14290-bib-0050] Muly E. C. , Maddox M. and Smith Y. (2003) Distribution of mGluR1alpha and mGluR5 immunolabeling in primate prefrontal cortex. J. Comp. Neurol. 467, 521–535. 14624486 10.1002/cne.10937

[jnc14290-bib-0051] Murase S. , Mathé J. M. , Grenhoff J. and Svensson T. H. (1993) Effects of dizocilpine (MK‐801) on rat midbrain dopamine cell activity: differential actions on firing pattern related to anatomical localization. J. Neural Transm. Gen. Sect. 91, 13–25. 8452684 10.1007/BF01244915

[jnc14290-bib-0052] Murphy E. , Dalley J. and Robbins T. (2005) Local glutamate receptor antagonism in the rat prefrontal cortex disrupts response inhibition in a visuospatial attentional task. Psychopharmacology 179, 99–107. 15678364 10.1007/s00213-004-2068-3

[jnc14290-bib-0053] Musante V. , Neri E. , Feligioni M. *et al* (2008) Presynaptic mGlu1 and mGlu5 autoreceptors facilitate glutamate exocytosis from mouse cortical nerve endings. Neuropharmacology 55, 474–482. 18625255 10.1016/j.neuropharm.2008.06.056 PMC3310906

[jnc14290-bib-0054] Oliver Y. P. , Ripley T. L. and Stephens D. N. (2009) Ethanol effects on impulsivity in two mouse strains: similarities to diazepam and ketamine. Psychopharmacology 204, 679–692. 19263039 10.1007/s00213-009-1500-0

[jnc14290-bib-0055] Paine T. A. , Tomasiewicz H. C. , Zhang K. and Carlezon W. A., Jr (2007) Sensitivity of the five‐choice serial reaction time task to the effects of various psychotropic drugs in sprague‐dawley rats. Neural Mech. Personal. Disord. Alcohol. Addict. 62, 687–693. 10.1016/j.biopsych.2006.11.017 17343834

[jnc14290-bib-0056] Park Y.‐K. , Galik J. , Ryu P. D. and Randic M. , (2004). Activation of presynaptic group I metabotropic glutamate receptors enhances glutamate release in the rat spinal cord substantia gelatinosa. Neurosci. Lett., Festschrift dedicated to Prof. Manfred Zimmermann on the occasion of his 70th birthday 361, 220–224. 10.1016/j.neulet.2003.12.075 15135933

[jnc14290-bib-0057] Pozzi L. , Baviera M. , Sacchetti G. , Calcagno E. , Balducci C. , Invernizzi R. W. and Carli M. (2011) Attention deficit induced by blockade of N‐methyl D‐aspartate receptors in the prefrontal cortex is associated with enhanced glutamate release and cAMP response element binding protein phosphorylation: role of metabotropic glutamate receptors 2/3. Neuroscience 176, 336–348. 21193020 10.1016/j.neuroscience.2010.11.060

[jnc14290-bib-0058] Robbins T. (2002) The 5‐choice serial reaction time task: behavioural pharmacology and functional neurochemistry. Psychopharmacology 163, 362–380. 12373437 10.1007/s00213-002-1154-7

[jnc14290-bib-0059] Romano C. , Sesma M. A. , McDonald C. T. , O'malley K. , van den Pol A. N. and Olney J. W. , (1995). Distribution of metabotropic glutamate receptor mGluR5 immunoreactivity in rat brain. J. Comp. Neurol. 355, 455–469. https://doi.org/10.1002/cne.903550310 7636025 10.1002/cne.903550310

[jnc14290-bib-0060] Rosenbrock H. , Kramer G. , Hobson S. , Koros E. , Grundl M. , Grauert M. , Reymann K. G. and Schröder U. H. (2010) Functional interaction of metabotropic glutamate receptor 5 and NMDA‐receptor by a metabotropic glutamate receptor 5 positive allosteric modulator. Metabotropic Glutamate Recept. Cogn. 639, 40–46. 10.1016/j.ejphar.2010.02.057 20371241

[jnc14290-bib-0061] Shigemoto R. , Nomura S. , Ohishi H. , Sugihara H. , Nakanishi S. and Mizuno N. (1993) Immunohistochemical localization of a metabotropic glutamate receptor, mGluR5, in the rat brain. Neurosci. Lett. 163, 53–57. 8295733 10.1016/0304-3940(93)90227-c

[jnc14290-bib-0062] Stefani M. R. and Moghaddam B. (2010) Activation of type 5 metabotropic glutamate receptors attenuates deficits in cognitive flexibility induced by NMDA receptor blockade. Eur. J. Pharmacol. 639, 26–32. 20371234 10.1016/j.ejphar.2010.01.028 PMC3359134

[jnc14290-bib-0063] Suzuki Y. , Jodo E. , Takeuchi S. , Niwa S. and Kayama Y. (2002) Acute administration of phencyclidine induces tonic activation of medial prefrontal cortex neurons in freely moving rats. Neuroscience 114, 769–779. 12220577 10.1016/s0306-4522(02)00298-1

[jnc14290-bib-0064] Thomas L. S. , Jane D. E. , R. Harris J. and Croucher M. J. , (2000). Metabotropic glutamate autoreceptors of the mGlu5 subtype positively modulate neuronal glutamate release in the rat forebrain in vitro. Neuropharmacology 39, 1554–1566. 10854900 10.1016/s0028-3908(99)00223-3

[jnc14290-bib-0065] Tsai P. J. , Wu J. P. , Lin N. N. , Kuo J. S. and Yang C. S. (1996) In vivo, continuous and automatic monitoring of extracellular ascorbic acid by microdialysis and on‐line liquid chromatography. J. Chromatogr. B Biomed. Appl. 686, 151–156. 8971595 10.1016/s0378-4347(96)00224-1

[jnc14290-bib-0066] Urwyler S. (2011) Allosteric modulation of family C G‐protein‐coupled receptors: from molecular insights to therapeutic perspectives. Pharmacol. Rev. 63, 59–126. 21228259 10.1124/pr.109.002501

[jnc14290-bib-0067] Wang M. and Arnsten A. F. T. (2015) Contribution of NMDA receptors to dorsolateral prefrontal cortical networks in primates. Neurosci. Bull. 31, 191–197. 25754145 10.1007/s12264-014-1504-6 PMC4734117

