# Supplementary material for: Bidirectional variation in glutamate efflux in the medial prefrontal cortex induced by selective positive and negative allosteric mGluR5 modulators
Source: J Neurochem. 2018 Feb 12;145(2):111–24. doi: 10.1111/jnc.14290 (PMC5972455; doi:10.1111/jnc.14290)
Supplement: Supplementary file 1 [file JNC-145-111-s001.pptx]

## Slide 1
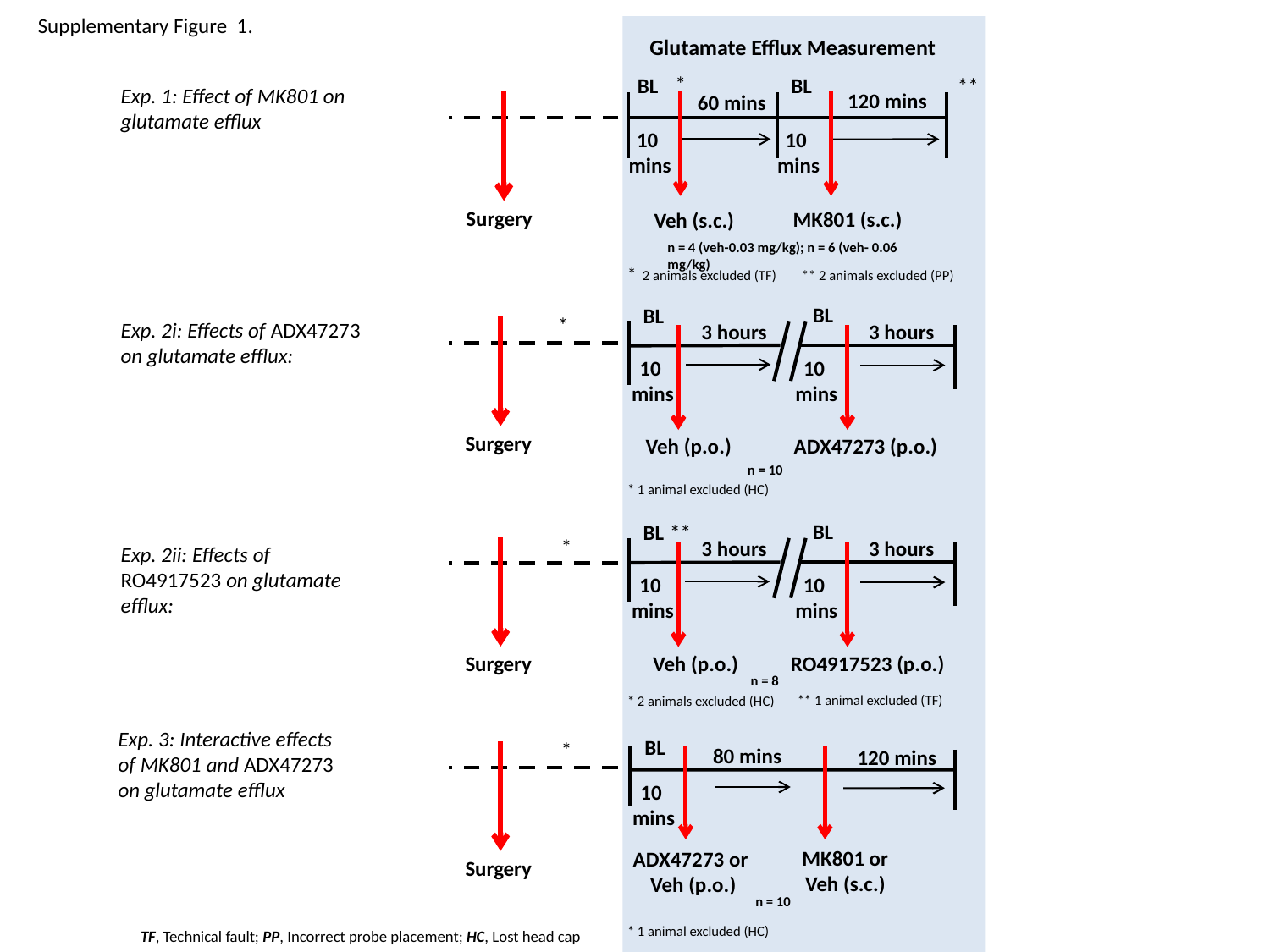

Supplementary Figure 1.
Glutamate Efflux Measurement
*
BL
120 mins
60 mins
10
mins
10
mins
 MK801 (s.c.)
Veh (s.c.)
BL
**
Exp. 1: Effect of MK801 on glutamate efflux
Surgery
n = 4 (veh-0.03 mg/kg); n = 6 (veh- 0.06 mg/kg)
* 2 animals excluded (TF) ** 2 animals excluded (PP)
BL
BL
3 hours
3 hours
10
mins
10
mins
Veh (p.o.)
ADX47273 (p.o.)
*
Exp. 2i: Effects of ADX47273 on glutamate efflux:
Surgery
n = 10
* 1 animal excluded (HC)
BL
BL
3 hours
3 hours
10
mins
10
mins
Veh (p.o.)
RO4917523 (p.o.)
**
*
Exp. 2ii: Effects of RO4917523 on glutamate efflux:
Surgery
n = 8
** 1 animal excluded (TF)
* 2 animals excluded (HC)
Exp. 3: Interactive effects of MK801 and ADX47273 on glutamate efflux
BL
80 mins
120 mins
10
mins
MK801 or Veh (s.c.)
ADX47273 or Veh (p.o.)
*
Surgery
n = 10
* 1 animal excluded (HC)
TF, Technical fault; PP, Incorrect probe placement; HC, Lost head cap
