# Supplementary material for: Bidirectional variation in glutamate efflux in the medial prefrontal cortex induced by selective positive and negative allosteric mGluR5 modulators
Source: J Neurochem. 2018 Feb 12;145(2):111–24. doi: 10.1111/jnc.14290 (PMC5972455; doi:10.1111/jnc.14290)
Supplement: Supplementary file 2 — Figure S1. Bidirectional variation in glutamate efflux in the medial prefrontal cortex induced by selective positive and negative allosteric mGluR5 modulators. Sarah N. Isherwood, Trevor W. Robbins, Jeffrey W. Dalley, Anton Pekcec. Figure S2. Schematic diagram illustrating the experimental timeline for each experiment; n numbers represent the total number of rats used in each experiment (i.e. before exclusion); exclusion reason included: TF, technical fault; PP, incorrect probe placement; HC, lost head cap. [file JNC-145-111-s002.pdf]

Supplementary Figure 1.

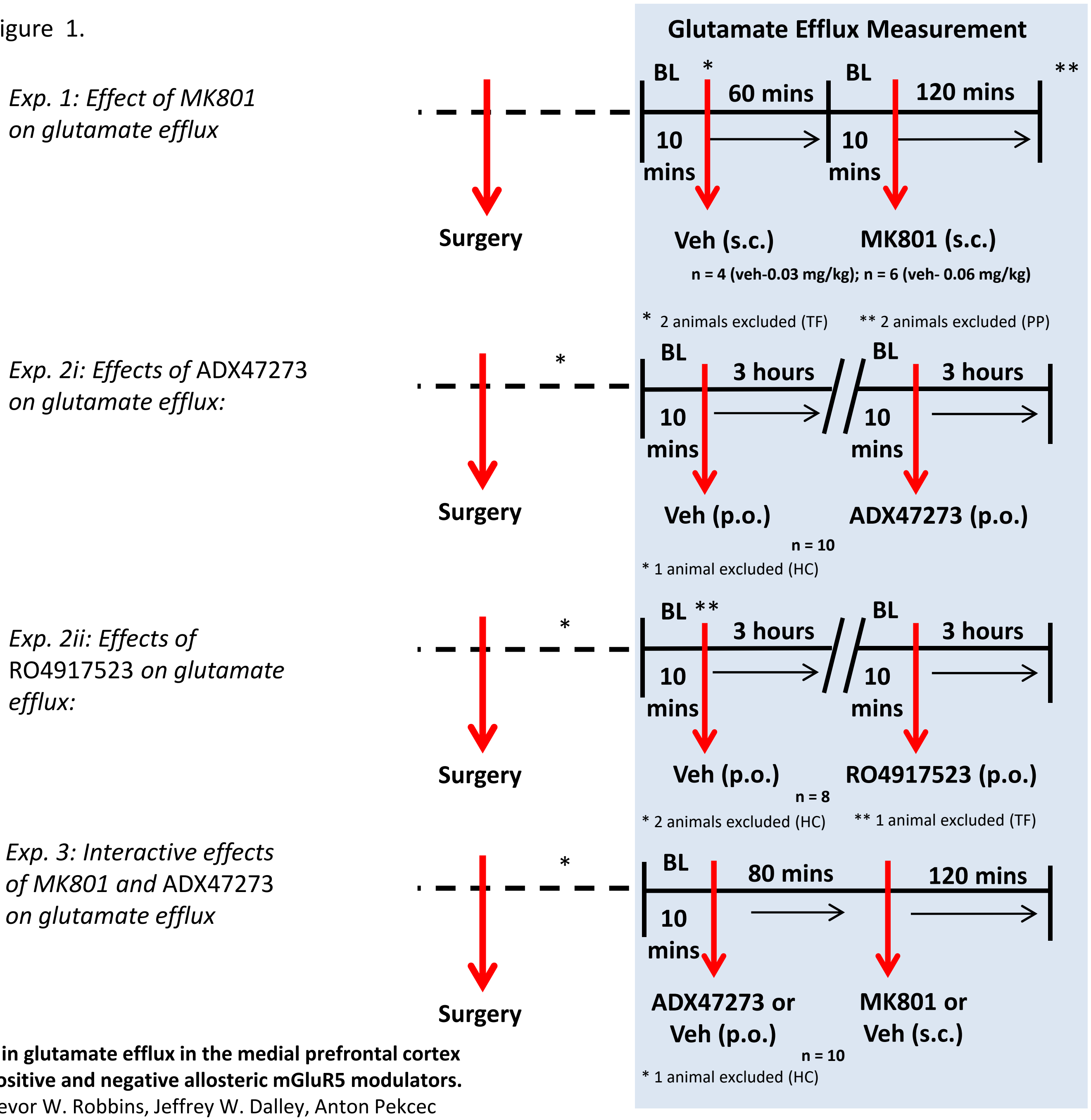

Bidirectional variation in glutamate efflux in the medial prefrontal cortex induced by selective positive and negative allosteric mGluR5 modulators.

Sarah N. Isherwood, Trevor W. Robbins, Jeffrey W. Dalley, Anton Pekcec

Schematic diagram illustrating the experimental timeline for each experiment; n numbers represent the total number of rats used in each experiment (i.e. before exclusion); exclusion reason included: *TF*, technical fault; *PP*, incorrect probe placement; *HC*, lost head cap.

Supplementary Figure 2.

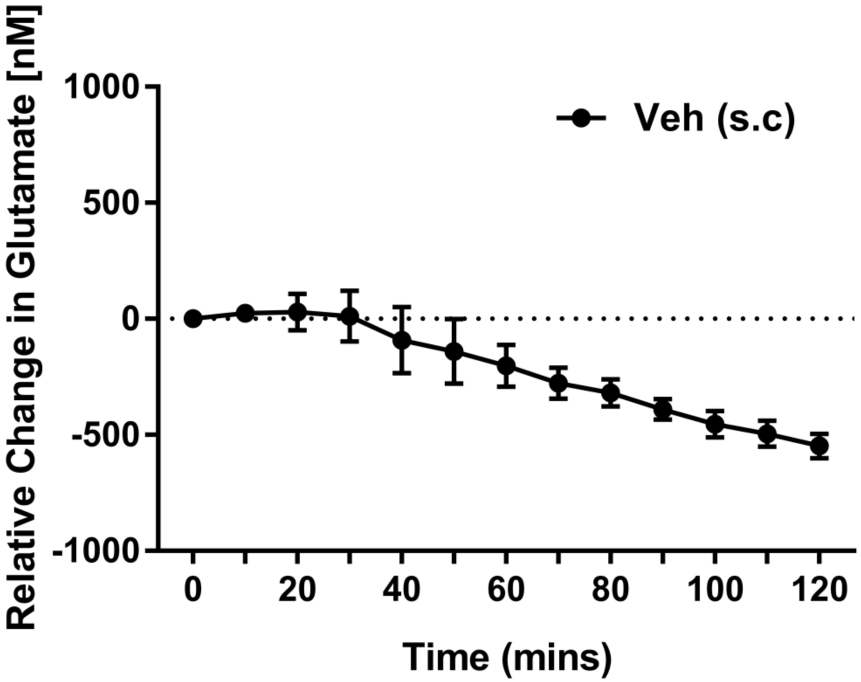

Effect of vehicle treatment on glutamate efflux in the mPFC. Rats received vehicle (s.c) 80 minutes into experiment 3 (n = 10 rats). Data points represent mean ± SEM.
